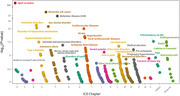# Polygenic score‐wide association study of comorbidities to incident dementia

**DOI:** 10.1002/alz.095150

**Published:** 2025-01-09

**Authors:** Sang‐Hyuk Jung, Jakob Woerner, Dong‐gi Lee, Yonghyun Nam, Li Shen, Hong‐Hee Won, Dokyoon Kim

**Affiliations:** ^1^ Perelman School of Medicine, University of Pennsylvania, Philadelphia, PA USA; ^2^ Samsung Advanced Institute for Health Sciences and Technology (SAIHST), Sungkyunkwan University, Samsung Medical Center, Seoul Korea, Republic of (South); ^3^ Institute for Biomedical Informatics, University of Pennsylvania, Philadelphia, PA USA

## Abstract

**Background:**

Previous studies have reported various prodromal symptoms and comorbidities that precede the development of dementia. In this study, we comprehensively investigate the impact of genetic predispositions to multiple comorbidities on the risk of incident dementia.

**Methods:**

Our study included 377,653 participants of European descent from the UK Biobank, comprising 370,183 controls and 7,470 cases of incident dementia. We utilized large‐scale genome‐wide association study (GWAS) data from the FinnGen Consortium to generate the polygenic score (PGS) for quantifying genetic predispositions. From 2,405 GWASs, we selected those with a heritability greater than 1% using LD Score regression. We then analyzed pairwise genetic correlations, retaining the more heritable factor in cases where the collinearity (genetic correlation) exceeded 0.9. Consequently, we included 331 eligible PGSs in the Cox proportional hazard model‐based polygenic score‐wide association study (PolyWAS) for incident dementia.

**Results:**

We identified 53 significant comorbidity‐PGSs (Bonferroni corrected p‐value = 0.05/331) through PolyWAS analysis, adjusted for the covariates age, sex, *ApoE* ε4 status, and dementia PGS. Notably, cardiometabolic‐related diseases categorized under the ICD‐10 code blocks ‘I’ (diseases of the circulatory system) and ‘E’ (endocrine, nutritional and metabolic diseases) showed the most significant associations with the risk of incident dementia. Depression and mood disorders under category ‘F’ (mental, behavioral and neurodevelopmental disorders) were risk factors in Alzheimer’s disease (AD) but not in Vascular dementia (VD). In addition, the genetic predisposition to Medical abortion under the category ‘O’ emerged as a risk factor for all‐cause dementia and its subtypes, including AD and VD. All significant PGSs were positively associated with incident dementia.

**Conclusion:**

Our study implemented a novel association test approach using comorbidity‐PGSs to investigate the genetic predispositions associated with numerous comorbidities and their impact on incident dementia and its subtypes. The proposed PolyWAS approach can better advance our knowledge of disease mechanisms and enhance our understanding of the shared genetics of comorbidities.